# Cardiorespiratory effects of spontaneous breathing in two different models of experimental lung injury: a randomized controlled trial

**DOI:** 10.1186/cc7108

**Published:** 2008-11-04

**Authors:** Dirk Varelmann, Thomas Muders, Jörg Zinserling, Ulf Guenther, Anders Magnusson, Göran Hedenstierna, Christian Putensen, Hermann Wrigge

**Affiliations:** 1 Department of Anesthesiology and Intensive Care Medicine, University of Bonn, Sigmund-Freud-Strasse 25, D-53105 Bonn, Germany; 2 Department of Radiology, University of Uppsala, University Hospital, SE-75185 Uppsala, Sweden; 3 Department of Clinical Physiology, University of Uppsala, University Hospital, SE-75185 Uppsala, Sweden

## Abstract

**Introduction:**

Acute lung injury (ALI) can result from various insults to the pulmonary tissue. Experimental and clinical data suggest that spontaneous breathing (SB) during pressure-controlled ventilation (PCV) in ALI results in better lung aeration and improved oxygenation. Our objective was to evaluate whether the addition of SB has different effects in two different models of ALI.

**Methods:**

Forty-four pigs were randomly assigned to ALI resulting either from hydrochloric acid aspiration (HCl-ALI) or from increased intra-abdominal pressure plus intravenous oleic acid injections (OA-ALI) and were ventilated in PCV mode either with SB (PCV + SB) or without SB (PCV – SB). Cardiorespiratory variables were measured at baseline after induction of ALI and after 4 hours of treatment (PCV + SB or PCV – SB). Finally, density distributions and end-expiratory lung volume (EELV) were assessed by thoracic spiral computed tomography.

**Results:**

PCV + SB improved arterial partial pressure of oxygen/inspiratory fraction of oxygen (PaO_2_/FiO_2_) by a reduction in intrapulmonary shunt fraction in HCl-ALI from 27% ± 6% to 23% ± 13% and in OA-ALI from 33% ± 19% to 26% ± 18%, whereas during PCV – SB PaO_2_/FiO_2 _deteriorated and shunt fraction increased in the HCl group from 28% ± 8% to 37% ± 17% and in the OA group from 32% ± 12% to 47% ± 17% (*P *< 0.05 for interaction time and treatment, but not ALI type). PCV + SB also resulted in higher EELV (HCl-ALI: 606 ± 171 mL, OA-ALI: 439 ± 90 mL) as compared with PCV – SB (HCl-ALI: 372 ± 130 mL, OA-ALI: 192 ± 51 mL, with *P *< 0.05 for interaction of time, treatment, and ALI type).

**Conclusions:**

SB improves oxygenation, reduces shunt fraction, and increases EELV in both models of ALI.

## Introduction

Alveolar recruitment in response to therapeutic interventions such as mechanical ventilation with positive end-expiratory pressure (PEEP) has been suggested to differ between direct (pulmonary) or indirect (extrapulmonary) acute lung injury (ALI) or the acute respiratory distress syndrome (ARDS) [1-3]. In direct ALI/ARDS, the injury originates from the alveolar epithelium and is characterized by alveolar collapse, fibrinous exudates, and alveolar wall edema [4], which might result in an increased lung elastance while chest wall elastance is often normal Computed tomography (CT) scans show equal amounts of consolidation and ground-glass opacities, with consolidated areas favoring the vertebral regions [5]. In indirect ALI/ARDS, the insult originates from the vascular endothelium and may cause less damage to the lung but may be associated with increased chest wall elastance [6] often caused by restricted movements and cranial shift of the diaphragm due to increased intra-abdominal pressure (IAP) [1,7]. Ground-glass opacity predominates and is evenly distributed [5]. Thus, direct and indirect ALI/ARDS have been suggested to have two distinct diseases with different respiratory mechanics, histopathology, and CT findings [1,5,8,9].

Maintaining unsupported spontaneous breathing (SB) with airway pressure release ventilation (APRV) has been shown to improve oxygenation when compared with controlled mechanical ventilation in patients with ALI/ARDS of different origin [10,11]. SB counteracts atelectasis formation and favors alveolar recruitment [12,13], resulting in an improvement in ventilation/perfusion (${\dot{V}}_{A}/\dot{Q}$) matching [14-17]. On the other hand, during controlled ventilation, as the diaphragm relaxes, it is displaced by the weight of the contents of the abdominal cavity, leading to the redistribution of tidal volumes (V_T_) to anterior, non-dependent, and less perfused lung regions [13,18]. These effects may be even more pronounced in indirect ALI/ARDS. Whether previously shown beneficial cardiopulmonary effects of SB might differ depending on ALI/ARDS origin has not been investigated yet. We asked the question of whether SB during pressure-controlled ventilation (PCV) improves oxygenation, ${\dot{V}}_{A}/\dot{Q}$ distribution, shunt fraction, and end-expiratory lung volume (EELV) in two different models of ALI. This research question was tested in porcine models of hydrochloric acid (HCl)-induced ALI and in the combination of oleic acid (OA) injection and elevated IAP.

## Materials and methods

### Animals

Experiments were approved by the animal ethics committee of the University of Uppsala. Forty-four pigs were anesthetized and mechanically ventilated in the supine position. The animals of each group were further randomly assigned into subgroups receiving either PCV with SB (PCV + SB) or without SB (PCV – SB). Anesthesia, tracheotomy, and fluid infusion were performed as previously described [12]. A detailed description of measurements and statistical analysis is provided in Additional data file 1.

### Ventilatory setting

#### Pressure-controlled ventilation without spontaneous breathing

PCV is a time-cycled ventilatory mode applied at a respiratory rate (RR) of 15 breaths per minute, an inspiratory to expiratory (I/E) ratio of 1:1, an inspiratory fraction of oxygen (FiO_2_) of 0.5, a PEEP of 5 cm H_2_O, and an inspiratory pressure (P_insp_) resulting in a V_T _of approximately 10 mL/kg using a standard ventilator (Servo I; Siemens-Elema AB, Solna, Sweden) to maintain normocapnia (35 mm Hg < arterial partial pressure of carbon dioxide [PaCO_2_] < 45 mm Hg). P_insp _was adjusted accordingly. SB efforts were excluded by the absence of negative deflections in the esophageal pressure (P_es_) tracings. After induction of ALI (baseline ALI [BL-ALI]), RR had to be increased as well as P_insp _to compensate for a decrease of compliance and to maintain normocapnia. I/E, PEEP, and FiO_2 _were kept constant. After BL-ALI measurements, the animals were randomly assigned to continue controlled mechanical ventilation or to resume SB.

#### Pressure-controlled ventilation with spontaneous breathing

Ventilator settings were guided by the principles described above. RR was decreased to 15 breaths per minute, which corresponds to approximately 50% of the RR after induction of ALI (BL-ALI), for re-institution of SB (confirmed by animal-generated inspiratory flow and concomitant negative P_es _deflections). I/E ratio was kept constant.

### Lung injury

#### Hydrochloric acid-induced acute lung injury

HCl (0.1 M) was intratracheally instilled until a stable lung injury was achieved.

#### Oleic acid-induced acute lung injury

The abdominal pressure was increased to 20 cm H_2_O by infusion of normal saline into the abdominal cavity, followed by central venous injection of OA. We aimed at a target arterial partial pressure of oxygen (PaO_2_)/FiO_2 _of less than 200 mm Hg, but a PaO_2_/FiO_2_of less than 300 mm Hg was accepted after stabilization of ALI.

### Measurements

Instrumentation of the animals has been described previously [19]. Heart rate (HR) and intravascular pressures were measured using standard technology [19]. Cardiac output (CO) and intrathoracic blood volume (ITBV) were determined with the transpulmonary thermal-indicator dilution technique [19]. Systemic and pulmonary vascular resistances were calculated using standard equations. Gas flow and derived variables, as well as airway and P_es _values, were continuously determined and stored on personal computers for offline analyses. Blood gases were analyzed using standard blood gas electrodes, and oxygen saturation and hemoglobin were analyzed using spectrophotometry. ${\dot{V}}_{A}/\dot{Q}$ distribution was measured using the multiple inert gas elimination technique (MIGET) [20]. Spiral scans were performed at the end of the experiments for determination of density distributions and pulmonary air content, which should represent EELV. Scans were carried out in randomized directions at end-inspiration and end-expiration with the tube clamped, and images were stored on personal computers for offline analysis.

### Protocol

**Figure 1:**
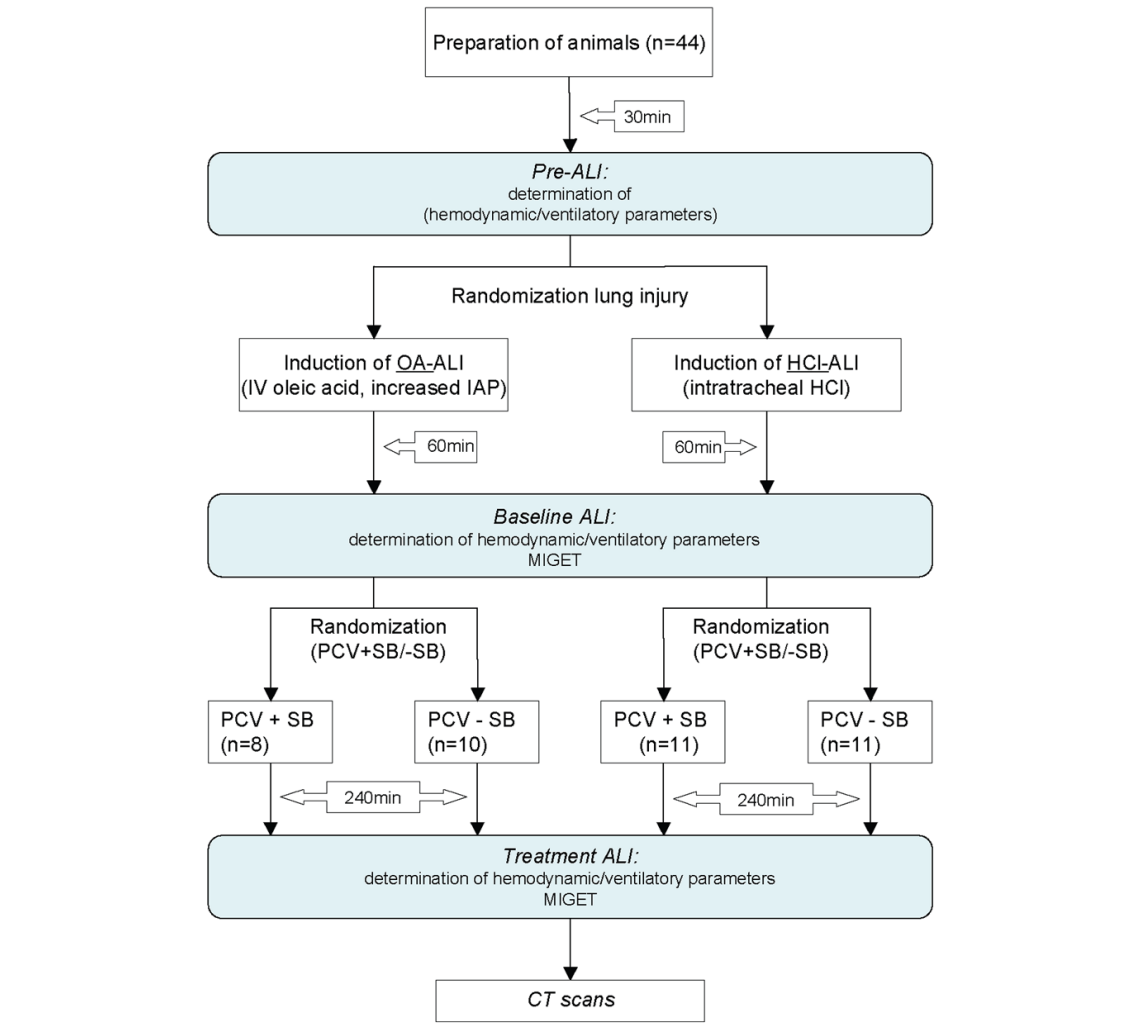
Flowchart of the study protocol. The grey boxes represent the measurement points. ALI, acute lung injury; CT, computed tomography; HCl, hydrochloric acid; HCl-ALI, hydrochloric acid-induced acute lung injury; IAP, intra-abdominal pressure; IV, intravenous; MIGET, multiple inert gas elimination technique; OA-ALI, oleic acid-induced acute lung injury (combined with an increased intra-abdominal pressure); PCV + SB, pressure-controlled ventilation with spontaneous breathing; PCV – SB, pressure-controlled ventilation without spontaneous breathing.

An illustration of the study protocol is given in Figure 1. In brief, blood gases and hemodynamic and ventilatory parameters were obtained 30 minutes after completing instrumentation (Pre-ALI) and 60 minutes after completing initiation of ALI (BL-ALI), together with the first MIGET measurement, and the animals were subjected to controlled mechanical ventilation without SB. Thereafter, animals of the two groups (HCl-induced and OA-induced ALI) were further randomly assigned either to continue with controlled mechanical ventilation (PCV – SB) or to additional SB (PCV + SB). After 240 minutes, another set of measurements, including MIGET and CT scans, was performed (Treatment). The overall study period was 8 hours. Four animals died in the course of the experiments: two pigs died directly after induction of lung injury; in two others, for technical reasons, no CT scans were obtained, resulting in n = 11 in the HCl-ALI PCV + SB group, n = 11 in the HCl-ALI PCV – SB group, n = 8 in the OA-ALI PCV + SB group, and n = 10 in the OA-ALI PCV – SB group.

### Statistical analysis

To detect differences in PaO_2_/FiO_2_, shunt fraction, EELV, and amount of non-aerated lung between the ventilatory setting and lung injury groups with the given parallel design at a significance level of 5% (α = 0.05) with a probability of 80% (β = 0.20) based on an estimated difference of 0.62 of the mean standard deviation (SD) of the parameter, the number of animals to be studied is at least 40. Results are expressed as mean ± SD, and all analyses were performed using a statistical software package (Statistica for Windows 6.0; StatSoft, Inc., Tulsa, OK, USA). Data were tested for normal distribution by the Shapiro-Wilks W test and analyzed by a two-way analysis of variance for repeated measurements with factors 'mode' and 'time'. When a significant F ratio was obtained, differences between the means were isolated for the specific factor (and for all factors in case of significant interaction) with the *post hoc *Tukey multiple comparison test. Differences were considered to be statistically significant for *P *values of less than 0.05.

## Results

### Lung injury

Induction of ALI led to a comparable and severe hypoxemia with PaO_2_/FiO_2 _below 200 mm Hg in 38 out of 40 animals in both HCl-ALI and OA-ALI (Table S1 in Additional data file 1). As expected by the study design, in the HCl group, respiratory system compliance was decreased mainly by decreased lung compliance, and, in OA-ALI, due to decreased chest wall compliance associated with increased abdominal pressure (Table 1). Thus, in HCl-ALI, mean transpulmonary airway pressure (P_transp, mean_) was higher at all times after induction of ALI (*P *< 0.05), and the dynamic intrinsic PEEP (PEEP_I, dyn_) was not influenced by the type of injury (Table 1). In both models, RR and airway pressures (Table 1) had to be increased to maintain alveolar ventilation (minute ventilation [V_E_]) after ALI induction. In the OA group, EELV and longitudinal lung dimensions (distances of apex – dome and apex – costodiaphragmatic recessus) were significantly smaller than in the HCl group (*P *< 0.05, Table S4 in Additional data file 1). In HCl-ALI, shunt decreased after 4 hours of treatment (*P *< 0.05, Table 2), whereas dead space ventilation (${\dot{V}}_{A}/\dot{Q}$ → ∞) increased irrespective of ALI type and ventilatory mode (*P *< 0.05, effect time).

**Table 1:** Ventilation and respiratory system mechanics

		SB	Baseline ALI	Treatment	Lung injury	Time	Injury type	Mode	Inter-action
RR, breaths per minute	HCl	+	28.2 ± 3.4	45.3 ± 8.5^b^	^a^	^a^		^a^	
		-	28.4 ± 2.8	28.3 ± 3.2^c^					
	OA	+	29.2 ± 0.1	43.5 ± 6.7^b^					
		-	29.1 ± 1.8	29.2 ± 1.7^c^					
V_T_, mL	HCl	+	326 ± 46	212 ± 28^b^					
		-	303 ± 23	272 ± 20^b^	^a^	^a^		^a^	TM^a^
	OA	+	317 ± 41	190 ± 19^b^					
		-	285 ± 52	260 ± 31^b^					
V_T, sb_, mL	HCl	+	n/a	135 ± 20			^a^		
		-	n/a	n/a					
	OA	+	n/a	95 ± 19^d^					
		-	n/a	n/a					
V_E_, liters	HCl	+	8.8 ± 1.1	8.6 ± 1.5	^a^	^a^		^a^	
		-	8.5 ± 0.9^c^	7.6 ± 1.1^c^					
	OA	+	9.1 ± 1.2	7.7 ± 1.2					
		-	8.0 ± 1.0^c^	7.4 ± 0.6^c^					
PaCO_2_, mm Hg	HCl	+	40 ± 6	45 ± 9	^a^	^a^		^a^	TM^a^
		-	42 ± 9	54 ± 13^c^					
	OA	+	41 ± 9	43 ± 10					
		-	46 ± 10	54 ± 17^c^					
P_transp, mean_, mbar	HCl	+	5.5 ± 3.0	5.5 ± 4.6	^a^	^a^	^a^		
		-	6.8 ± 3.3	8.1 ± 3.4					
	OA	+	2.1 ± 3.0	2.7 ± 3.4					
		-	0.5 ± 3.2	3.1 ± 3.5					
PEEP_I, dyn_, mbar	HCl	+	0.0 ± 1.1	0.3 ± 0.3					
		-	0.7 ± 0.6	0.9 ± 0.9					
	OA	+	0.0 ± 1.6	0.8 ± 0.5					
		-	0.3 ± 0.3	0.0 ± 1.6					
C_cw_, mL/mbar	HCl	+	89.3 ± 39.3	n/a			^a^		
		-	96.8 ± 34.9	115.5 ± 64.3					
	OA	+	40.9 ± 14.8^d^	n/a					
		-	39.3 ± 11.2^d^	49.5 ± 27.6^d^					
C_lung_, mL/mbar	HCl	+	19.5 ± 4.0	n/a	^a^		^a^		
		-	16.4 ± 8.4	13.5 ± 2.8					
	OA	+	21.3 ± 4.7^d^	n/a					
		-	21.7 ± 8.3^d^	16.2 ± 7.9^d^					
R, mbar/L per second	HCl	+	7.0 ± 0.7^b^	n/a			^a^	^a^	
		-	7.5 ± 1.6^b^	8.5 ± 2.8					
	OA	+	8.5 ± 1.0^b^	n/a					
		-	9.0 ± 3.0^b^	11.9 ± 2.7					
EELV, mL	HCl	+		606 ± 171			^a^	^a^	
		-		372 ± 130^c^					
	OA	+		439 ± 90^d^					
		-		192 ± 51^c, d^					

Pre-acute lung injury (ALI) (Table S2 in additional data file 1) was tested only against baseline ALI. *Post hoc *testing was always performed if a significant F ratio for a factor or the interaction of factors was obtained by repeated measures analysis of variance (^a^*P *< 0.05), but only significant differences are marked: ^b^*P *< 0.05 for within-group differences (ALI versus Treatment), ^c^*P *< 0.05 for between-group differences (PCV + SB versus PCV – SB), and ^d^*P *< 0.05 for between-group differences (HCl-ALI versus OA-ALI) (*post hoc *Tukey multiple comparison test). +, pressure-controlled ventilation with maintained spontaneous breathing; -, pressure-controlled ventilation without spontaneous breathing; C_cw_, chest wall compliance; C_lung_, lung compliance; EELV, end-expiratory lung volume; HCl, hydrochloric acid-induced acute lung injury; M, mode; n/a, not applicable; OA, oleic acid-induced acute lung injury; PaCO_2_, arterial partial pressure of carbon dioxide; PCV + SB, pressure-controlled ventilation with spontaneous breathing; PCV – SB, pressure-controlled ventilation without spontaneous breathing; PEEP_I, dyn_, dynamic intrinsic positive end-expiratory pressure; P_transp, mean_, mean transpulmonary airway pressure; R, respiratory system resistance; RR, respiratory rate; SB, spontaneous breathing; T, time; V_E_, minute ventilation; V_T_, tidal volume; V_T, sb_, tidal volume of spontaneous breaths.

**Table 2:** Oxygenation and hemodynamic parameters

		SB	BL-ALI	Treatment	Lung injury	Time	Injury type	Mode	Inter-action
PaO_2_/FiO_2_, mm Hg	HCl	+	132 ± 18	150 ± 50	^a^				TM^a^
		-	151 ± 58	137 ± 104					
	OA	+	145 ± 51	184 ± 116					
		-	146 ± 68	109 ± 46					
HR, beats per minute	HCl	+	96 ± 12	112 ± 11	^a^	^a^			
		-	100 ± 22	108 ± 20					
	OA	+	102 ± 11	110 ± 23					
		-	112 ± 18	119 ± 23					
MAP, mm Hg	HCl	+	79 ± 8	86 ± 9^b^		^a^	^a^		
		-	77 ± 23	92 ± 13^b^					
	OA	+	93 ± 12^d^	97 ± 13^b, d^					
		-	101 ± 10^d^	104 ± 15^b, d^					
CVP, mm Hg	HCl	+	11 ± 2	10 ± 2^b^	^a^				
		-	12 ± 3	12 ± 2^b^					
	OA	+	15 ± 2^d^	14 ± 2^b, d^					
		-	15 ± 4^d^	14 ± 3^b, d^					
SVR, dyne-second/cm^5^	HCl	+	1,335 ± 198	1,057 ± 191^b^	^a^	^a^	^a^		
		-	1,255 ± 429	1,072 ± 333^b^					
	OA	+	1,513 ± 344	1,281 ± 388^b^					
		-	1,490 ± 384	1,060 ± 206^b^					
CO, L/minute	HCl	+	4.1 ± 0.3	4.6 ± 0.8	^a^			^a^	
		-	4.2 ± 0.9^c^	4.8 ± 0.9^c^					
	OA	+	4.2 ± 0.7	4.3 ± 0.8					
		-	4.8 ± 0.8^c^	5.5 ± 0.7^c^					
DO_2_, mL/minute	HCl	+	323 ± 28	393 ± 71	^a^	^a^			
		-	336 ± 63	369 ± 89					
	OA	+	335 ± 89	360 ± 48					
		-	408 ± 80	430 ± 118					
VO_2_, mL/minute	HCl	+	181 ± 31	172 ± 33	^a^		^a^		
		-	169 ± 23	172 ± 32					
	OA	+	159 ± 27^c^	147 ± 34^c^					
		-	154 ± 32^c^	167 ± 42^c^					
Shunt ${\dot{V}}_{A}/\dot{Q}$ < 0.005, %Q_T_	HCl	+	27.1 ± 6.2	23.3 ± 12.7		^a^			TM^a^
		-	27.7 ± 7.9	37.4 ± 17.4^b^					
	OA	+	32.6 ± 18.9	26.0 ± 17.9					
		-	32.4 ± 12.4	47.2 ± 17.1^b^					
Dead space ${\dot{V}}_{A}/\dot{Q}$ > 100, %Ve	HCl	+	33.0 ± 5.5	45.1 ± 11.8		^a^			
		-	34.4 ± 5.9	38.7 ± 3.9					
	OA	+	39.1 ± 6.6	44.9 ± 12.8					
		-	39.0 ± 6.0	46.2 ± 12.2					

Pre-acute lung injury (ALI) (Table S1 in additional data file) was tested only against baseline ALI (BL-ALI). *Post hoc *testing was always performed if a significant F ratio for a factor or the interaction of factors was obtained by repeated measures analysis of variance (^a^*P *< 0.05), but only significant differences are marked: ^b^*P *< 0.05 for within-group differences (BL-ALI versus Treatment), ^c^*P *< 0.05 for between-group differences (HCl-ALI versus OA-ALI), and ^d^*P *< 0.05 for between-group differences (PCV + SB versus PCV – SB) (*post hoc *Tukey multiple comparison test). +, pressure-controlled ventilation with maintained spontaneous breathing; -, pressure-controlled ventilation without spontaneous breathing; CO, cardiac output; CVP, central venous pressure; DO_2_, oxygen delivery; HCl, hydrochloric acid-induced acute lung injury; HCl-ALI, hydrochloric acid-induced acute lung injury; HR, heart rate; M, mode; MAP, mean arterial pressure; OA, oleic acid-induced acute lung injury; OA-ALI, oleic acid-induced acute lung injury; PaO_2_/FiO_2_, arterial partial pressure of oxygen/inspiratory fraction of oxygen; PCV + SB, pressure-controlled ventilation with spontaneous breathing; PCV – SB, pressure-controlled ventilation without spontaneous breathing; %Q_T_, percentage of cardiac output; SB, spontaneous breathing; SVR, systemic vascular resistance; T, time; ${\dot{V}}_{A}/\dot{Q}$, ventilation/perfusion (ratio); %Ve, percentage of minute ventilation; VO_2_, oxygen consumption.

**Figure 2:**
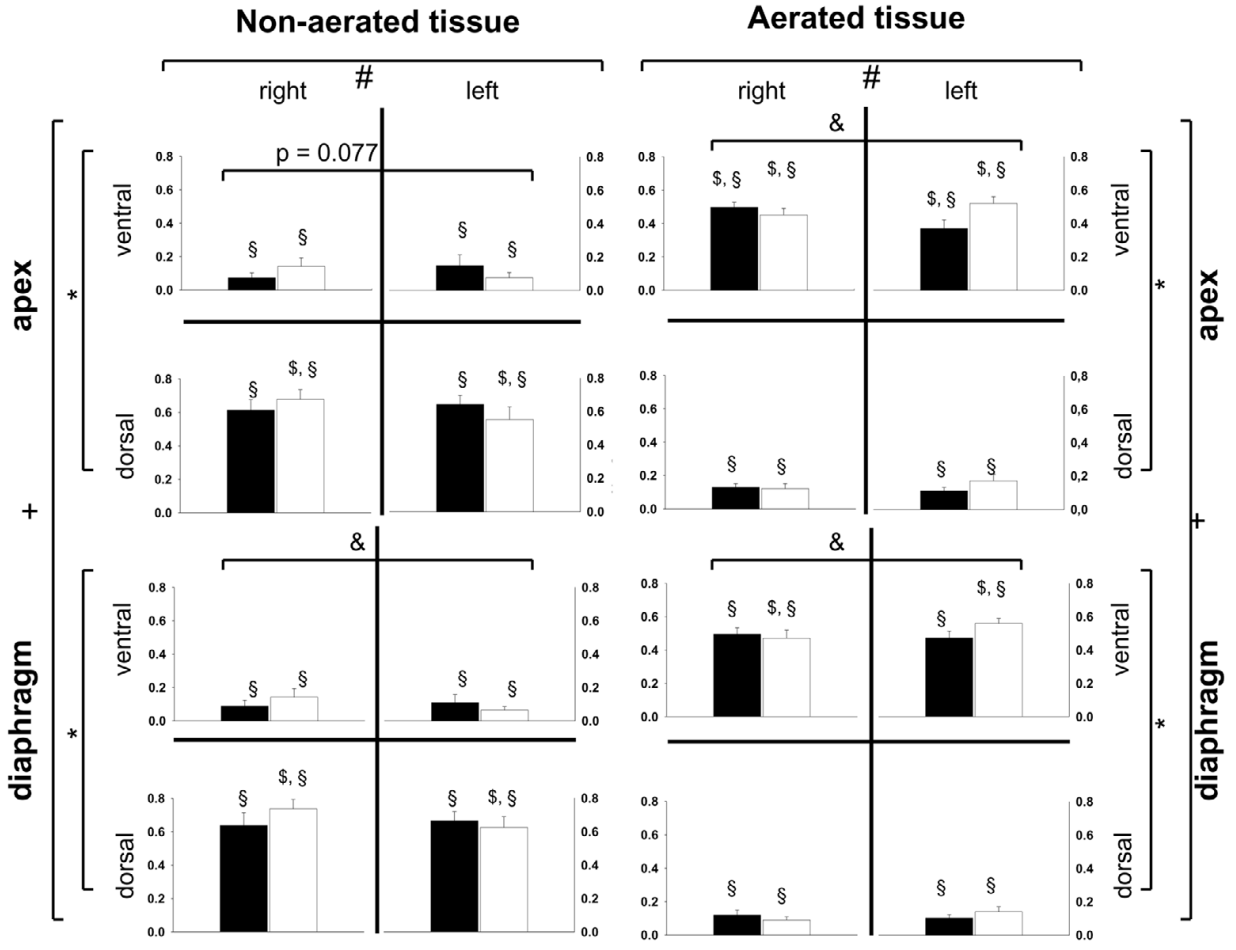
Distribution of fractions of non-aerated and aerated tissue in end-expiratory spiral computed tomography scans. Filled bars indicate oleic acid-induced acute lung injury (ALI), and outlined bars indicate hydrochloric acid-induced ALI. Fractions of densities are presented as mean ± standard error of the mean. *P < 0.05: ventral versus dorsal, analysis of variance (ANOVA). ^+^*P *< 0.05: interaction of ventral-dorsal and apical-diaphragmatic distribution, ANOVA. ^#^*P *< 0.05: interaction injury and left-right distribution. ^&^*P *< 0.05: left versus right in juxtadiaphragmatic regions for hydrochloric acid-induced ALI, Tukey's honest significant differences (HSD). ^§^*P *< 0.05: apex versus diaphragm for corresponding region of interest (ROI), Tukey's HSD. ^$^*P *< 0.05: left versus right for corresponding ROI, Tukey's HSD.

For both types of ALI, the CT scans showed a gravity-dependent distribution of non-aerated tissue, predominantly in the dorsal areas (*P *< 0.05), and the aerated tissue found in the ventral parts of the lung (*P *< 0.05) (Figure 2). This effect is more pronounced in the juxtadiaphragmatic lung regions (*P *< 0.05) compared with the apical parts of the lung and is not dependent on the ALI type. The shunt fraction determined with the MIGET correlates with the amount of non-aerated lung tissue observed in the spiral CT scans (HCl-ALI: y = 0.85 x - 0.02, *R*^2 ^= 0.58; OA-ALI: y = 1.19 x - 0.03, *R*^2 ^= 0.84). In HCl-generated ALI, however, the amount of non-aerated tissue is increased in the right region of interest (ROI) (*P *< 0.05), whereas an increase in aeration is found in the left ROI (*P *< 0.05). The SD of non-aerated tissue (SD_atelect_) and the fraction of non-aerated tissue per ROI (SD%_atelect_) over all slices of the spiral scans did not differ between the two models of ALI (SD_atelect_: 4.3 versus 3.9; SD%_atelect_: 0.13 versus 0.13, for HCl-induced versus OA-induced ALI).

### Pressure-controlled ventilation without spontaneous breathing

**Figure 3:**
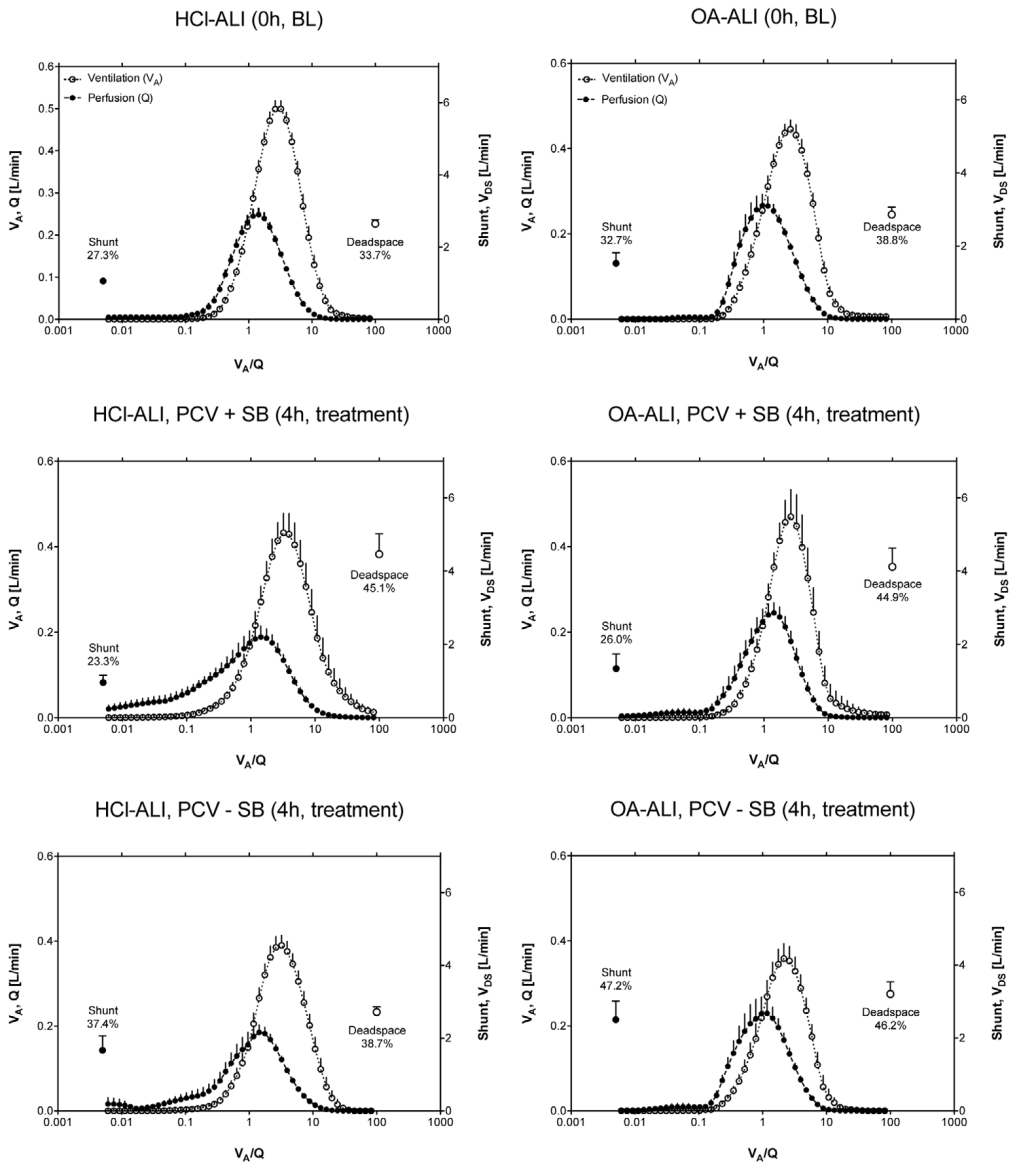
Ventilation/perfusion distributions. Continuous distributions of ventilation and blood flow (mean ± standard error of the mean) plotted versus ventilation/perfusion ratio (${\dot{V}}_{A}/\dot{Q}$). BL indicates baseline measurement after induction of stable acute lung injury, and treatment indicates measurement after 4 hours of pressure-controlled ventilation (PCV) either with (+ SB) or without (- SB) spontaneous breathing. HCl-ALI, hydrochloric acid-induced acute lung injury; OA-ALI, oleic acid-induced acute lung injury; V_DS_, deadspace ventilation.

In PCV – SB, PaO_2_/FiO_2 _deteriorated significantly (*P *< 0.05 for interaction of time and ventilatory mode) (Table 2). CT scans showed a greater fraction of non-aerated tissue in this group (*P *< 0.05, Figure 2). V_T _decreased slightly as compared with baseline ALI (*P *< 0.05), whereas PaCO_2 _increased (*P *< 0.05) despite higher mean airway (P_aw, mean_) and transpulmonary (P_transp, mean_) pressures (*P *< 0.05) (Table 1). CO increased during the 4-hour treatment period in this group (*P *< 0.05) (Table 2), and a marked increase in blood flow to shunt regions (${\dot{V}}_{A}/\dot{Q}$ = 0) (*P *< 0.05, Table 2) with a reduction in blood flow to regions with a normal ${\dot{V}}_{A}/\dot{Q}$ (0.1 <${\dot{V}}_{A}/\dot{Q}$ < 10) was observed (Figure 3).

### Pressure-controlled ventilation with spontaneous breathing

**Figure 4:**
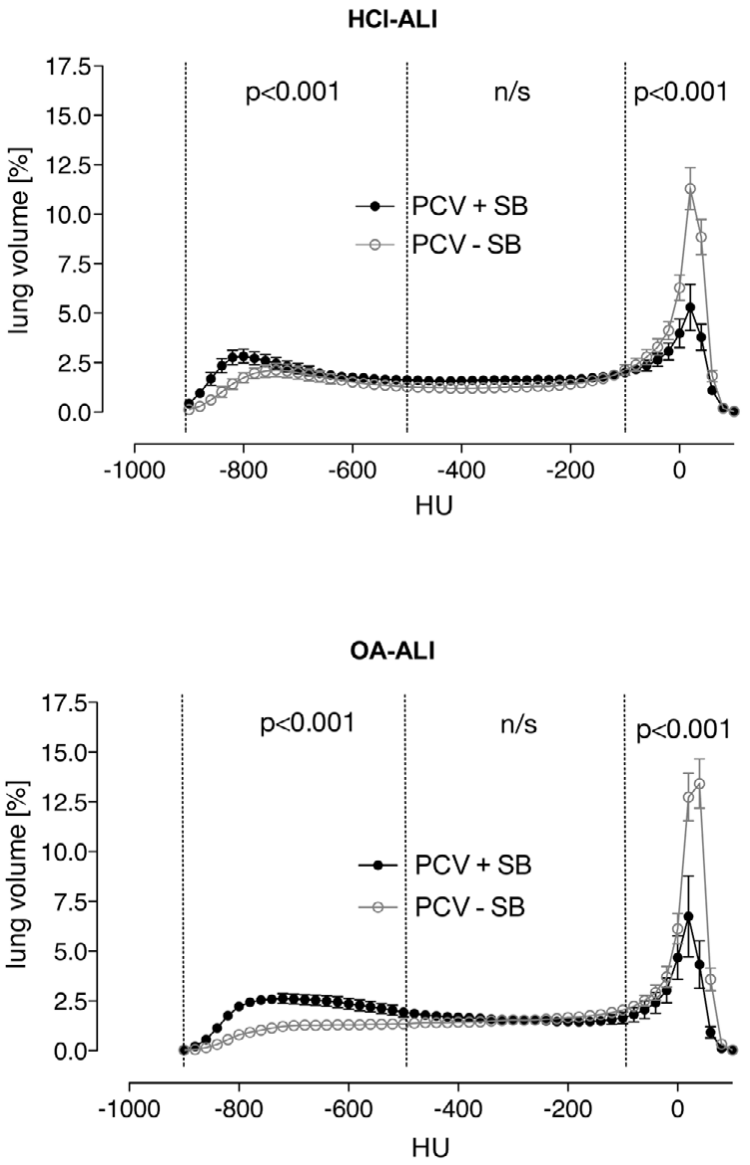
Density distributions. Density histograms taken from end-expiratory spiral computed tomography of all animals show normalized lung volume in Hounsfield units (HU) ranging from -1,000 to 100 plotted as mean ± standard error of the mean. Aeration categories (hyperinflated, normally aerated, poorly aerated, and non-aerated) are marked and were statistically compared (see Results and Discussion sections of the text for details) between pressure-controlled ventilation with (PCV + SB) and without (PCV – SB) spontaneous breathing. There were no significant differences caused by the type of acute lung injury. HCl-ALI, hydrochloric acid-induced acute lung injury; n/s, not significant; OA-A.

PCV + SB improved PaO_2_/FiO_2 _during 4 hours of treatment (*P *< 0.05, interaction time course and ventilatory mode, Table 2). Overall lung density was lower compared with PCV – SB (*P *< 0.05); accordingly, the fraction of normally aerated tissue was higher in the PCV + SB group (*P *< 0.05) (Figure 4). The EELV and longitudinal lung dimensions were greater during SB compared with the PCV – SB group (*P *< 0.05). These effects were independent of the ALI type, with EELV and longitudinal dimensions always greater in HCl-ALI. SB led to an increase in RR (*P *< 0.05) with a concomitant decrease in VT (*P *< 0.05) and increases in V_E _and PaCO_2_. The increase in PaCO_2_, however, was lower as compared with PCV – SB (*P *< 0.05, Table 1). The V_T _of spontaneous breaths was lower in the OA-ALI group. The increases in P_aw, mean _and P_transp, mean _(*P *< 0.05) were comparable with the increases in the PCV – SB group, PEEP was comparable in the two groups, and PEEP_I, dyn _was not significantly different between the two groups and was less than 1 cm H_2_O. Blood flow to low ${\dot{V}}_{A}/\dot{Q}$ compartments (0.005 <${\dot{V}}_{A}/\dot{Q}$ < 0.1) increased during PCV + SB in the HCl group only (*P *< 0.001, Table S5 in Additional data file 1). In both groups, PCV – SB and PCV + SB, the HR and mean arterial pressure (MAP) increased during the 4-hour treatment period (*P *< 0.05), whereas central venous pressure (CVP) and systemic vascular resistance (SVR) dropped (*P *< 0.05, Table 2), and pulmonary artery occlusion pressure (PAOP) and ITBV remained unchanged (Table S3 in Additional data file 1).

## Discussion

Our data confirm previous findings that SB during PCV leads to an improvement in oxygenation through the reduction in shunt and restoration of aeration in previously non-aerated lung regions. These effects are not influenced by the type of ALI/ARDS studied here.

### Lung injury

Although one should be careful in drawing conclusions from findings in animal models for treatment of patients with ARDS, our different lung injury types mimic relevant aspects of the clinical situation. HCl aspiration damaged the alveolar epithelium and increased lung elastance usually due to alveolar flooding and collapse, reduced removal of edema fluid, and reduced production of surfactant [4,21-23]. Commonly, HCl-induced ALI is regarded as a form of direct ALI. OA injection combined with abdominal hypertension [1] causes damage to the vascular endothelium, resulting in increased chest wall elastance usually associated with microvascular congestion, interstitial edema, and recruitment of inflammatory cells, whereas the intra-alveolar spaces are spared [24], mimicking indirect ALI. Although OA exhibits direct toxicity to endothelial cells [25], the elicited lung injury might not be similar to ALI caused by sepsis. However, OA generates a reproducible injury within a reasonable time frame.

According to our knowledge, the differences of direct and indirect ALI/ARDS have been described qualitatively only, revealing a heterogeneous distribution pattern (for example, 'patchy pattern') of normal lung, regions with ground-glass opacity, and consolidated areas. In the current literature, different distribution patterns of inhomogeneities are described [2,5,26,27]. We attempted to quantify the heterogeneities by determining the SD of density distributions in eight ROIs per transverse slide assessed with spiral CT scans. However, this approach did not reveal any quantitative differences and the authors were not able to distinguish the type of injury by visual inspection in a significant number of animals. This suggests either that there are no morphological differences between these models of ALI or that the differences are too small to be detected with the used CT technique. Desai and colleagues [8] were not able to describe a single CT feature to predict whether ARDS in humans is of direct or indirect origin. These findings suggest that both injury types result in interstitial pulmonary edema as a common final path. The greater amount of injury in the right lungs in HCl-induced ALI is well known from aspiration pneumonia.

The additional fluid volume infused into the abdominal cavity in the OA group influences hemodynamic parameters; MAP was higher in the OA group (*P *< 0.05, effect injury type) as an effect of an increased SVR (*P *< 0.05, effect injury type; Table 2), and CO was not different between the injury models. However, the ITBV was not significantly different between OA-induced and HCl-induced ALI (Table W3 in Additional data file 1), and on average very little normal saline had to be replaced for maintaining IAP ( < 100 mL), thus effects other than intravascular shifting of intraperitoneal fluid might account for this.

The rationale to investigate the effects of SB in two different ALI models was that they might differ in their potential for recruitment [1,28,29]. Recruitment maneuvers differ in their effect on oxygenation and lung mechanics in an animal model of intratracheal and intraperitoneal lipopolysaccharide injections, with recruitment maneuvers being more effective in animals with intraperitoneally injected lipopolysaccharide [29]. Recent data, however, challenged this concept: a multicenter CT study in 68 patients with ALI or ARDS was unable to detect any difference in alveolar recruitment potential depending on the type of ALI, but huge individual differences were detected [30]. A recent study found the volume recruited by different levels of PEEP (10 and 14 cm H_2_O) in patients with direct and indirect ARDS to be similar, but classification of ARDS was uncertain in more than one third (37%) of patients [31]. The PEEP used in this study was considerably low and might not have prevented atelectasis formation. The aim of this study, however, was to study the effects of SB in different ALI models and not the effects of other recruitment strategies such as recruitment maneuvers or high PEEP. Intrinsic PEEP was below 1 cm H_2_O in all situations and therefore was not considered clinically significant. The meta-analysis of studies did not find any differences in outcome in patients with direct or indirect ALI/ARDS [32]. These recent findings suggest that differences in alveolar recruitment potential are attributable to individual differences between patients rather than to the systematic origin of ALI/ARDS. This is in line with our experimental findings that beneficial effects of SB on lung recruitment do not depend on the origin of ALI/ARDS.

### Effects of spontaneous breathing on respiratory variables

PCV + SB resulted in a higher EELV, greater lung dimensions, and less non-aerated tissue (Figure 4), indicating that SB prevents a loss of aeration. During SB, the posterior muscular sections of the diaphragm move more than the anterior tendon plate [17] and ventilation is shifted to the dependent lung regions [33], thereby counteracting atelectasis formation and resulting in improvement in ${\dot{V}}_{A}/\dot{Q}$ matching [14,16]. The finding that EELV was lower in OA-induced ALI can be explained by the elevated IAP and, as a consequence, a cranial displacement of the diaphragm with compression atelectasis or consolidation of the juxtadiaphramatic lung regions [34,35].

V_T _tended to be smaller when SB was maintained. This is a consequence of the unsupported spontaneous breaths, which occurred on the lower pressure level only. The spontaneous V_T _(V_Tsb_) was lower in the OA group due to the more cranially displaced diaphragm compared with the HCl group. As spontaneous breaths coincided with mechanical breaths delivered by the ventilator, it is difficult to determine the V_T _solely generated by ventilator. With the high spontaneous RR on the lower pressure level, plausible 'ventilator V_T_' could not be calculated.

The good correlation of the shunt fraction determined with the MIGET with the amount of non-aerated lung tissue observed in the spiral CT scans has already been shown by others [5]. This suggests that loss of aeration (also indicated by the reduction in EELV) was the main reason for the shunt fraction and that the prevention of this loss of aeration in these lung areas by SB contributed to the improvement in oxygenation, regardless of ALI type. This is in agreement with previous studies reporting a reduction in intrapulmonary shunting in PCV with SB [10,12,16,36,37]. Intrapulmonary shunt in ARDS/ALI has been found to correlate directly with the quantity of non-aerated tissue in dependent lung regions [5,14,38]. In HCl-induced ALI with maintained SB, the blood flow to low ${\dot{V}}_{A}/\dot{Q}$ (0.005 <${\dot{V}}_{A}/\dot{Q}$ < 0.1) was significantly higher than in HCl-ALI without SB and in OA-ALI with and without SB. HCl instillation led to alveolar flooding and collapse, and the physiologic response is to divert blood flow away from non-ventilated regions (hypoxic pulmonary vasoconstriction). PCV + SB in HCl-induced ALI might have restored ventilation in those regions and might have led to an increase in perfused low ${\dot{V}}_{A}/\dot{Q}$ areas that participate in gas exchange. The effects of low ${\dot{V}}_{A}/\dot{Q}$ on blood oxygenation, however, will depend on FiO_2_. With low FiO_2_, low ${\dot{V}}_{A}/\dot{Q}$ regions contribute to impaired oxygenation, but at high FiO_2 _there will be no substantial effect. High FiO_2 _will more easily cause collapse (atelectasis) of the low ${\dot{V}}_{A}/\dot{Q}$ regions. The deterioration in oxygenation in the PCV – SB group can be explained by the reduced blood flow to normal ${\dot{V}}_{A}/\dot{Q}$ (0.1 <${\dot{V}}_{A}/\dot{Q}$ < 10) and the concomitant increase in shunt after 4 hours of treatment. The greater dispersion of blood flow (logSD_Q_) in HCl-induced lung injury after 4 hours of treatment might indicate damage that is more severe [39]. However, this does not translate into a greater deterioration of oxygenation. SB, on the other hand, had no effect on the dispersion of ventilation distribution. Thus, impairments in oxygenation in the PCV – SB group are caused by the increase in shunt. All animals showed a unimodal distribution of perfusion and ventilation, and the residual sum of squares (RSS) was exceptionally low, indicating adequate MIGET data [39].

### Effects of spontaneous breathing on hemodynamic parameters

In contrast to previously published data [10-13,16,36,37], we observed an increase in CO during PCV – SB over the 4-hour treatment period. An animal study found less depression of CO and oxygen delivery (DO_2_) with PCV + SB compared with PCV at similar transpulmonary pressures [40]. In our study, the CO during PCV + SB and PCV – SB was comparable to previously published studies [12,16]., and the more pronounced increase in the PCV – SB group does not lead to a significant increase in DO_2_. This suggests that the higher CO was required to maintain an adequate DO_2_. Linear regression analysis revealed a reasonable inverse correlation between PaO_2_/FiO_2 _and CO during PCV + SB (*r*^2 ^= 0.42, *P *< 0.05) after the 4-hour treatment period compared with *r*^2 ^= 0.23 (*P *< 0.05) in the PCV – SB group. The change in CO is positively correlated with the increase in intrapulmonary shunt (*r*^2 ^= 0.36), which is in line with previously conducted studies [41]. However, as the time frame of our experiments was relatively short, one has to be careful to extrapolate the results on patients with impaired CO due to, for example, septic cardiomyopathy. The increase in CO after 4 hours of treatment in the controlled mechanical ventilation group might also be explained by the increased PaCO_2 _and the lower pH; also, the latter was not statistically significant. In the OA group subjected to controlled mechanical ventilation, the drop in SVR is especially pronounced, which might explain the highest CO in this group. The ITBV indicates comparable volume status. However, the CVP and the MAP were higher in the indirect ALI group, most likely due to elevated IAP [7,42,43]. The mode of ventilation did not influence oxygen consumption (VO_2_), which is in line with previous observations [10,14], whereas VO_2 _was significantly lower in the OA-induced ALI group, most likely as a consequence of impaired organ perfusion due to increased abdominal pressure.

In this study, we used time-cycled PCV with suppression of SB compared with PCV with maintained SB (also termed airway pressure release ventilation [APRV] or biphasic positive airway pressure [bi-level]). Currently, a number of ventilatory modes supporting SB are available and have been used in animal models of ALI. Noisy pressure support ventilation improved oxygenation and reduced venous admixture at a lower P_aw, mean _compared with PCV in a model of surfactant depletion [44]. On comparing PCV with different modes of assisted SB (biphasic positive airway pressure, pressure-controlled assisted ventilation, and pressure support ventilation) in a saline lavage animal model, assisted SB was found to be superior to PCV in terms of oxygenation and hemodynamic parameters [45]. The transpulmonary pressure was different in our groups as a result of the increased IAP in the OA group and the comparable PEEP in both groups. Increasing the PEEP to match the groups for transpulmonary pressure could have influenced the results, but this was beyond our scope to investigate the effects of SB. Although several studies reported beneficial effects of SB in ALI, we were able to show that PCV with maintained SB has positive effects regardless of the model of ALI used in our study.

## Conclusion

Although the different origin of the ALI results in different respiratory mechanics, EELV, hemodynamic parameters, and shunt fraction, PCV with SB improved oxygenation, reduced shunt fraction, and restored EELV in both types of ALI.

## Key messages

• In pigs with different types of experimental acute lung injury, spontaneous breathing during airway pressure release ventilation leads to an improvement in oxygenation by a reduction in shunt fraction.

• Spontaneous breathing during pressure-controlled ventilation restores aeration in previously non-aerated lung regions, independently of the type of lung injury.

• The cardiac output during pressure-controlled ventilation with suppressed spontaneous breathing is higher than with maintained spontaneous breathing, and oxygen delivery is unaffected.

## Abbreviations

ALI: acute lung injury; APRV: airway pressure release ventilation; ARDS: acute respiratory distress syndrome; BL-ALI: baseline acute lung injury; CO: cardiac output; CT: computed tomography; CVP: central venous pressure; DO_2_: oxygen delivery; EELV: end-expiratory lung volume; FiO_2_: inspiratory fraction of oxygen; HCl: hydrochloric acid; HCl-ALI, hydrochloric acid-induced acute lung injury; HR: heart rate; IAP: intra-abdominal pressure; I/E: inspiratory/expiratory (ratio); ITBV: intrathoracic blood volume; MAP: mean arterial pressure; MIGET: multiple inert gas elimination technique; OA: oleic acid; OA-ALI, oleic acid-induced acute lung injury; PaCO_2_: arterial partial pressure of carbon dioxide; PaO_2_: arterial partial pressure of oxygen; P_aw, mean_: mean airway pressure; PCV: pressure-controlled ventilation; PEEP: positive end-expiratory pressure; PEEP_I, dyn_: dynamic intrinsic positive end-expiratory pressure; P_es_: esophageal pressure; P_insp_: inspiratory pressure; P_transp, mean_: mean transpulmonary airway pressure; ROI: region of interest; RR: respiratory rate; SB: spontaneous breathing; SD: standard deviation; SD_atelect_: standard deviation of non-aerated tissue; SD%_atelect_: fraction of non-aerated tissue per region of interest; SVR: systemic vascular resistance; ${\dot{V}}_{A}/\dot{Q}$: ventilation/perfusion (ratio); V_E_: minute ventilation; VO_2_: oxygen consumption; V_T_: tidal volume.

## Competing interests

The authors declare that they have no competing interests.

## Authors' contributions

DV participated in the design and coordination of the study, performed measurements, and wrote the manuscript. TM performed the CT analysis, participated in the study design, and helped draft the manuscript. JZ participated in the design of the study and performed measurements and the statistical analysis. UG analyzed data and helped draft the manuscript. AM participated in the study design and coordination and organized the CT measurements. GH participated in the design and coordination of the study and revised the manuscript. CP participated in the design and coordination of the study and helped draft the manuscript. HW designed and coordinated the study, performed measurements, and helped draft the manuscript. All authors edited and approved the final version of the manuscript.

## Supplementary Material

Additional data file 1 A Microsoft Word document giving a detailed description of the methods used for preparation of the animals, determination of ventilation-perfusion ratios, as well as analyses of computed tomography scans. Furthermore, tables providing additional data on oxygenation, respiratory system mechanics, and hemodynamic parameters are presented in this data file. Click here for file
