# Supplementary material for: Cardiorespiratory effects of spontaneous breathing in two different models of experimental lung injury: a randomized controlled trial
Source: Crit Care. 2008 Nov 4;12(6):R135. doi: 10.1186/cc7108 (PMC2646345; doi:10.1186/cc7108)
Supplement: Additional data file 1 — A Microsoft Word document giving a detailed description of the methods used for preparation of the animals, determination of ventilation-perfusion ratios, as well as analyses of computed tomography scans. Furthermore, tables providing additional data on oxygenation, respiratory system mechanics, and hemodynamic parameters are presented in this data file. [file cc7108-S1.doc]

##### Additional Data File 1

Cardiorespiratory effects of spontaneous breathing in two different models experimental lung injury

Dirk Varelmann^1^, Thomas Muders^1^, Jörg Zinserling^1^, Ulf Guenther^1^, Anders Magnusson^2^, Göran Hedenstierna^3^, Christian Putensen^1^, and Hermann Wrigge^1^.

^1^ Department of Anesthesiology and Intensive Care Medicine, University of Bonn, Sigmund-Freud-Strasse 25, D-53105 Bonn, Germany.

^2^ Department of Radiology, University of Uppsala, SE-75185 Uppsala, Sweden

^3^ Department of Clinical Physiology, University of Uppsala, SE-75185 Uppsala, Sweden

*Animals*

Animal experiments were performed in the laboratories of the Department of Clinical Physiology at Uppsala University Hospital, Uppsala, Sweden. 44 pigs (mixed breed of Hampshire, Yorkshire, and Swedish country breed, 29 ± 3 kg) were anesthetized and mechanically ventilated in supine position. Sealed envelopes were used to randomize pigs to either direct or indirect ALI, the sealed envelopes contained a second envelope, opened after completion of induction of ALI, to randomize the animals of each group to subgroups receiving either pressure controlled ventilation with (PCV + SB) or without SB (PCV - SB). Two pigs died directly after induction of lung injury, in two others no CT scans were obtained for technical reasons, resulting in n=11 in group direct ALI PCV + SB, n=11 in group direct ALI PCV - SB, n=8 indirect ALI PCV + SB, and n=10 in indirect ALI PCV - SB.

## *Anesthesia*

Anesthesia was induced with intramuscular atropine (0.04 mg/kg), tiletamin-zolazepam (6 mg/kg), and xylazine (2.2 mg/kg). Anesthesia was maintained by continuous infusion of ketamine (30 mg/kg/h), midazolam (0.1 mg/kg/h) and fentanyl (3.5 µg/kg/h) [12]. In the APRV - SB group pancuronium bromide (0.15 mg/kg/h) was added to suppress SB, verified by the absence of negative deflections on the esophageal pressure tracings. The animals were tracheotomized and ventilation was initiated via a cuffed endotracheal tube with an ID of 7 mm (Hi-Contour ® 107; Mallinckrodt Medical, Athlone, Ireland).

## *Lung Injury*

*Hydrochloric acid-induced injury:* For induction of HCl-ALI 0.1 M hydrochloric acid was intratracheally installed in portions of 30 mL (60-270 mL) until a stable lung injury judged by the decrease in PaO_2_/FiO_2_ below 300mmHg with a less than 10% difference in two subsequent blood gas analyses was achieved.

*Oleic acid-induced injury*: OA-ALI was achieved by an increase in abdominal pressure (IAP = 20 cmH_2_O) by infusion of warmed normal saline (2.5 – 5.5 L) into the abdominal cavity via an intraperitoneally inserted introducer sheath. This was followed by central venous injection of 0.1 mL/kg oleic acid suspended in 10 mL isotonic saline until a PaO_2_/FiO_2_ < 300mmHg was achieved. During injection blood pressure was stabilized with titrated doses of epinephrine. Intra-abdominal pressure was intermittently measured as bladder pressure and, if necessary, adjusted to maintain 20 cmH_2_O.

## *Ventilatory and Lung Mechanics Measurements*

Gas flow was measured at the proximal of the endotracheal tube using a heated pneumotachograph (Fleisch No 2; Fleisch, Lausanne, Switzerland), connected to a differential pressure transducer (Huba Control, Würenlos, Switzerland). Airway pressure (P_aw_) was measured at the proximal end of the endotracheal tube with another differential gas-pressure transducer (SMT, Munich, Germany). Esophageal pressure (P_es_) was measured with a balloon catheter (International Medical, Zutphen, The Netherlands) connected to differential pressure transducers (SMT, Munich, Germany). The validity of the esophageal balloon measurements in the supine animals was tested with the occlusion method [46, 47]. When the slope of the P_es_/P_aw_ tracing differed from 1, P_es_ was corrected according to Brunner and Wolff [48]. Intra-abdominal pressure (P_ab_) was measured intermittently in the urinary bladder after filling with 50mL of normal saline solution, as described previously [49]. All signals were sampled with an analog/digital converter board (PCM-DAS16S/12, Mansfield, MA, USA) and stored on a personal computer.

Tidal volume (V_T_) and minute ventilation (V_E_) were derived from the integrated gas flow signal and converted to body temperature pressure saturated (BTPS) conditions. Spontaneous breathing occurred on the lower pressure level only, the V_T_ of unsupported breaths, i.e. not coinciding with a change in pressure level initiated by the ventilator, was calculated. Respiratory rate (RR), inspiratory (T_i_) and expiratory (T_e_) times were determined from gas flow signal. Mean airway pressure was determined for each respiratory cycle, transpulmonary pressure was calculated as the difference of airway and esophageal pressure. The dynamic intrinsic PEEP (PEEP_I,dyn_) during controlled mechanical ventilation (PCV – SB) was measured as the difference between external PEEP and the airway pressure required to initiate an inspiratory flow at the transition from expiration to inspiration. During spontaneous breathing, PEEP_I,dyn_ corresponds to the absolute difference in esophageal pressure at end-expiration and the initiation of an inspiratory flow, after correction for time lags between flow and pressure tracings, detected by differences in timing between airway pressure decline and onset of expiratory flow during the transition from inspiration to expiration. All ventilatory variables were averaged over a 5 min period.

## *Gas Analysis, Ventilation-Perfusion Distribution*

Blood gases were measured using standard blood gas electrodes. Oxygen saturation and hemoglobin were analyzed using spectrophotometry.

Ventilation-perfusion ($\dot{V}_{A}/\dot{Q}$) distribution was measured using the multiple inert gas elimination technique (MIGET) [20]: six inert gases with different blood solubility coefficients are dissolved in isotonic saline and infused at a constant rate through a peripheral vein. Arterial and mixed venous blood samples are tonometered with gas and analyzed with an expired gas sample by gas chromatography (5890, series II, Hewlett-Packard). These data allow calculation of a virtually continuous $\dot{V}_{A}/\dot{Q}$ distribution against blood flow or ventilation. The different $\dot{V}_{A}/\dot{Q}$ regions are separated into shunt ($\dot{V}_{A}/\dot{Q}$ < 0.005) from regions of low $\dot{V}_{A}/\dot{Q}$ (0.005 < $\dot{V}_{A}/\dot{Q}$ < 0.1), normal $\dot{V}_{A}/\dot{Q}$ (0.1 < $\dot{V}_{A}/\dot{Q}$ < 10), high $\dot{V}_{A}/\dot{Q}$ (10 < $\dot{V}_{A}/\dot{Q}$ < 100), and deadspace ventilation ($\dot{V}_{A}/\dot{Q}$ > 100). Measures of $\dot{V}_{A}/\dot{Q}$ mismatch (dispersion) of perfusion and ventilation against $\dot{V}_{A}/\dot{Q}$ are expressed by standard deviation of the logarithmic distribution of perfusion () and ventilation ().

## *CT scanning and analysis for EELV determination*

A frontal topogram of the chest during ventilation was obtained with a Somatom Sensation 16 (Siemens, Erlangen, Germany). Guided by observation of airway pressure and flow curves, the tube was first clamped strictly at end-expiration immediately followed by a spiral scan (140 kV, 111 mA, 0.75 s for one spin resulting in an acquisition time of 15-20 s). A second spiral scan was performed with the tube clamped at end-inspiration. The direction of the spiral scans was randomized. CT images were transferred to a personal computer and analyzed (Osiris, University of Geneva, Geneva, Switzerland). The left and right lungs were chosen as region of interest (ROI) by drawing the external boundaries of the lungs at the inside of the ribs and the internal boundaries along the mediastinal organs. The left and right lungs were further divided into ventral and dorsal areas, resulting in four equivalent quadrants. Along the longitudinal axis extending from the tracheal bifurcation to the diaphragmatic dome, the lung was divided into equal ROIs (apical and diaphragmatic). Thus eight ROIs were be identified. The investigator (T. M.) was specially trained and blinded to ventilatory mode and lung injury. The number of pixels corresponding to each density value within the range of -1,000 to +100 Hounsfield units (HU) in the ROI of each slice were counted and stored by the software. Outliers (pixels > 100HU) constituted to less than 1% of all counts and were excluded.

Analysis included the following approaches:

1. The distances between lung apex and diaphragmatic dome including costodiaphragmatic recessus from end-expiratory spiral scan were measured.
2. Summarization of density counts in ROIs of all slices of the total spiral CT for analysis of continuous density distribution of the entire lungs and calculation of mean density. Four groups of density ranges with decreasing air content were defined as previously described [50-52] range I included densities between -1000 and -900 HU, previously defined as hyperinflation; range II included densities between -900 and -500 HU (normal aeration); range III included densities between -500 and -100 (poor aeration); and densities between -100 and 100 HU were considered as range IV, representing atelectasis or lung parenchyma with an air content of 10% or less.
3. The left and right lungs were further divided into a ventral, a medial ventral, a medial dorsal and a dorsal ROIs, resulting in eight ROIs per transversal slice (Figure S1). All slices of the spiral scans along the longitudinal axis from the tracheal bifurcation to the diaphragmatic dome were evaluated (Figure S2). The standard deviation of volume of non-aerated tissue (SD_atelect_) and fraction of non-aerated tissue per ROI (SD%_atelect_) was used as a substitute for density inhomogeneities.
4. Pulmonary air content was estimated as the sum of air content of each voxel (CT unit of volume) of the entire lung. Only voxels with densities with a range of -1,000 to -1 HU were included. Voxel size was calculated as the area of a pixel x 8 mm (thickness of slice) resulting in 1.96 ± 0.39 mm^3^. Given the know limitations (e. g. underestimation of gas volume due to the partial volume effect) [53], the resulting volume should correspond to EELV.

Figure Legends

Figure S1: Regions of Interest (ROI) for evaluation of density distribution heterogeneitiesFigure S1: Regions of Interest (ROI) for evaluation of density distribution heterogeneities

Left and right lungs were divided into 8 ROIs per transversal slide taken from the spiral computed tomography (CT) scans for determination of density distribution heterogeneities.

Figure S1: Regions of Interest (ROI) for evaluation of density distribution heterogeneities

**Table S1: Pre-ALI oxygenation and hemodynamic parameters**

|  |  | Pre-ALI |
| --- | --- | --- |
| PaO_2_/FiO_2_ | HCl | 516 ± 48 |
| [mmHg] | OA | 544 ± 56 |
| HR | HCl | 92 ± 20 |
| [bpm] | OA | 92 ± 15 |
| MAP | HCl | 84 ± 14 |
| [mmHg] | OA | 82 ± 12 |
| CVP | HCl | 9 ± 2 |
| [mmHg] | OA | 8 ± 2 |
| SVR | HCl | 1937 ± 419 |
| [dyne*s*cm^-5^] | HCl | 1908 ± 426 |
| CO | HCl | 3.2 ± 0.6 |
| [L/min] | OA | 3.2 ± 0.7 |
| DO_2_ | HCl | 306 ± 66 |
| [mL/min] | OA | 331 ± 86 |
| VO_2_ | HCl | 80 ± 17 |
| [mL/min] | OA | 77 ± 20 |

At Pre-ALI, pressure controlled ventilation with (PCV + SB) and without (PCV - SB) spontaneous breathing groups were taken together as both groups at this point were on PCV with suppression of SB and identical ventilatory settings.

HCl: hydrochloric acid-induced acute lung injury, OA: oleic acid-induced acute lung injury.

HR: heart rate, MAP: mean arterial pressure, CVP: central venous pressure,: mean pulmary arterial pressure, SVR: systemic vascular resistance, CO: cardiac output, DO_2_: oxygen delivery, VO_2_: oxygen consumption.

Table S2: Pre-ALI ventilatory and respiratory mechanics parameters

|  |  | Pre-ALI |
| --- | --- | --- |
| RR | HCl | 19.5 ± 1.2 |
| [min^-1^] | OA | 21.4 ± 4.6 |
| V_T_ | HCl | 345 ± 56 |
| [mL] | OA | 356 ± 67 |
| V_E_ | HCl | 6.7 ± 1.1 |
| [L] | OA | 7.3 ± 1.3 |
| PaCO_2_ | HCl | 40 ± 8 |
| [mmHg] | OA | 38 ± 7 |
| P_transp,mean_ | HCl | -0.2 ± 4.5 |
| [mbar] | OA | -0.7 ± 3.9 |
| C_cw_ | HCl | 93.3 ± 53.2 |
| [mL/mbar] | OA | 107.2 ± 47.5 |
| Cl_ung_ | HCl | 74.4 ± 44.4 |
| [mL/mbar] | OA | 73.4 ± 29.8 |
| R | HCl | 5.9 ± 1.4 |
| [mbar/L/s] | OA | 5.4 ± 1.3 |

At Pre-ALI, pressure controlled ventilation with (PCV + SB) and without (PCV - SB) spontaneous breathing groups were taken together as both groups at this point were on PCV with suppression of SB and identical ventilatory settings.

HCl: hydrochloric acid-induced acute lung injury, OA: oleic acid-induced acute lung injury.

RR: respiratory rate, V_T_: tidal volume, V_E_: minute ventilation, P_trans,mean_: mean transpulmonary airway pressure, C_cw_: chest wall compliance, C_lung_: lung compliance, R: respiratory system resistance

Table S3: Additional oxygenation and hemodynamic parameters

|  |  | SB | Pre-ALI | BL ALI | Treatment | Lung Injury | Time | Injury Type | Mode | Inter-action |
| --- | --- | --- | --- | --- | --- | --- | --- | --- | --- | --- |
| PAOP  [mmHg] | HCl | + | 8 ± 3 | 12 ± 5 | 14 ± 6 | * |  |  |  |  |
|  |  | - |  | 10 ± 3 | 10 ± 3 |  |  |  |  |  |
|  | OA | + | 8 ± 3 | 12 ± 4 | 11 ± 3 |  |  |  |  |  |
|  |  | - |  | 12 ± 5 | 12 ± 4 |  |  |  |  |  |
| MPAP | HCl | + | 19 ± 3 | 35 ± 5 | 33 ± 6 |  |  |  |  |  |
| [mmHg} |  | - |  | 35 ± 6 | 40 ± 5 |  |  |  |  |  |
|  | OA | + | 18 ± 3 | 37 ± 5 | 34 ± 5 |  |  |  |  |  |
|  |  | - |  | 39 ± 5 | 40 ± 8 |  |  |  |  |  |
| PVR | HCl | + | 286 ± 94 | 463 ± 81 | 353 ± 85 | * |  |  |  |  |
| [dyne*s*cm^-5^] |  | - |  | 491 ± 148 | 517 ± 94 |  |  |  |  |  |
|  | OA | + | 265 ± 103 | 469 ± 161 | 424 ± 97 |  |  |  |  |  |
|  |  | - |  | 470 ± 44 | 405 ± 78 |  |  |  |  |  |
| ITBV  [mL] | HCl | + | 508 ± 65 | 534 ± 124 | 525 ± 48 |  |  |  |  |  |
|  |  | - |  | 557 ± 47 | 540± 54 |  |  |  |  |  |
|  | OA | + | 553 ± 178 | 549 ± 92 | 545 ± 88 |  |  |  |  |  |
|  |  | - |  | 565 ± 133 | 650 ± 184 |  |  |  |  |  |
| Ven. Admix  [%] | HCl | + | 4.6 ± 1.8 | 22.1 ± 3.5 | 29.2 ± 12.0 | * | * |  |  | * TM |
|  |  | - |  | 22.5 ± 10.3 | 38.2 ± 21.3 +,# |  |  |  |  |  |
|  | OA | + | 6.3 ± 4.3 | 26.4 ± 17.8 | 29.6 ± 18.7 |  |  |  |  |  |
|  |  | - |  | 29.0 ± 16.8 | 45.0 ± 18.4 +,# |  |  |  |  |  |
| pH | HCl | + | 7.48 ± 0.08 | 7.45 ± 0.06 | 7.42 ± 0.07 | * |  |  |  |  |
|  |  | - |  | 7.45 ± 0.09 | 7.36 ± 0.09 |  |  |  |  |  |
|  | OA | + | 7.49 ± 0.08 | 7.39 ± 0.09 | 7.42 ± 0.09 |  |  |  |  |  |
|  |  | - |  | 7.37 ± 0.09 | 7.35 ± 0.12 |  |  |  |  |  |
| BE  [mmol/L] | HCl | + | 5.1 ± 2.0 | 3.6 ± 1.6 | 3.8 ± 1.6 | * | * |  | * | * TI, TM |
|  |  | - |  | 3.8 ± 2.0 | 3.2 ± 2.0 |  |  |  |  |  |
|  | OA | + | 5.0 ± 2.0 | -0.3 ± 2.3 § | 2.7 ± 2.0 § |  |  |  |  |  |
|  |  | - |  | 1.1 ± 2.3 § | 2.8 ± 2.2 +,§ |  |  |  |  |  |

Pre-ALI was only tested against Baseline (BL) Acute Lung Injury (ALI). At Pre-ALI pressure controlled ventilation with (PCV + SB) and without spontaneously breathing (PCV - SB) were taken together as both groups at this point were on PCV with suppression of SB and identical ventilatory settings.

HCl: hydrochloric acid-induced acute lung injury, OA: oleic acid-induced acute lung injury. SB: spontaneous breathing (+: pressure controlled ventilation with maintained spontaneous breathing, -: pressure controlled ventilation without SB). Interaction factors: T: time, M: mode, I: injury type.

PAOP: pulmonary arterial occlusion pressure, MPAP: mean pulmary arterial pressure, SVR: systemic vascular resistance, PVR: pulmonary vascular resistance, ITBV: intra-thoracic blood volume, ven. Admix: venous admixture, BE: base excess

*Post hoc* testing was always performed if a significant F ratio for a factor or the interaction of factors was obtained by repeated-measures analysis of variance (*: p < 0.05), but only significant differences are marked: +: p < 0.05 for within-group differences (BL ALI vs Treatment) and #: p < 0.05 for between-group differences (PCV + SB vs PCV - SB), §: p < 0.05 for between-group differences (HCl vs OA-ALI) (*post hoc* Tukey multiple comparison test).

### **Table S4**:Additional parameters of ventilation, respiratory system mechanics

|  |  |  | Pre-ALI | BL ALI | Treatment | Lung Injury | Time | Injury Type | Mode | Inter-action |
| --- | --- | --- | --- | --- | --- | --- | --- | --- | --- | --- |
| P_aw,mean_  [mbar] | HCl | + | 9.9 ± 0.4 | 16.1 ± 0.9 | 16.2 ± 2.2 | * | * |  |  |  |
|  |  | - |  | 16.6 ± 1.3 | 17.1 ± 2.4 |  |  |  |  |  |
|  | OA | + | 10.0 ± 0.3 | 16.4 ± 1.4 | 16.9 ± 1.8 |  |  |  |  |  |
|  |  | - |  | 16.1 ± 1.2 | 17.2 ± 1.8 |  |  |  |  |  |
| P_aw,max_  [mbar] | HCl | + | 15.8 ± | 28.8 ± 2.0 | 32.9 ± 4.1 | * | * |  |  | * TM |
|  |  | - |  | 30.2 ± 3.1 | 30.6 ± 4.8 |  |  |  |  |  |
|  | OA | + | 16.1 ± 0.9 | 29.8 ± 3.0 | 33.3 ± 6.4 |  |  |  |  |  |
|  |  | - |  | 28.6 ± 2.7 | 30.5 ± 3.7 |  |  |  |  |  |
| C_rs_  [mL/mbar] | HCl | + | 37.6 ± 7.5 | 16.0 ± 3.2 | n/a | * |  |  |  |  |
|  |  | - |  | 14.0 ± 3.0 | 12.1 ± 2.9 |  |  |  |  |  |
|  | OA | + | 39.5 ± 8.3 | 14.9 ± 3.0 | n/a |  |  |  |  |  |
|  |  | - |  | 14.0 ± 4.5 | 12.0 ± 3.3 |  |  |  |  |  |
| D(a-d)  [mm] | HCl | + |  |  | 147 ± 10 |  |  | * | * |  |
|  |  | - |  |  | 144 ± 6 |  |  |  |  |  |
|  | OA | + |  |  | 131 ± 8 § |  |  |  |  |  |
|  |  | - |  |  | 118 ± 7 §,+ |  |  |  |  |  |
| D(a-r) | HCl | + |  |  | 214 ± 14 |  |  |  |  |  |
| [mm] |  | - |  |  | 193 ± 12 + |  |  |  |  |  |
|  | OA | + |  |  | 188 ± 11 § |  |  |  |  |  |
|  |  | - |  |  | 161 ±12§,+ |  |  |  |  |  |

Pre-ALI was only tested against Baseline Acute Lung Injury (ALI). At Pre-ALI, pressure controlled ventilation with (PCV + SB) and without (PCV - SB) spontaneous breathing groups were taken together as both groups at this point were on PCV with suppression of SB and identical ventilatory settings.

HCl: hydrochloric acid-induced acute lung injury, OA: oleic acid-induced acute lung injury. SB: spontaneous breathing (+: pressure controlled ventilation with maintained spontaneous breathing, -: pressure controlled ventilation without SB). Interaction factors: T: time, M: mode, I: injury type.

P_aw,mean_: mean airway pressure, P_aw,max_: maximum airway pressure, C_rs_: respiratory system compliance, C_cw_: chest wall compliance, D(a-d): distance between lung apex and diaphragm, D(a-r): distance between lung apex and costodiaphragmatic recessus

*Post hoc* testing was always performed if a significant F ratio for a factor or the interaction of factors was obtained by repeated-measures analysis of variance (*: p < 0.05), but only significant differences are marked: +: p < 0.05 for within-group differences (ALI vs Treatment) and #: p < 0.05 for between-group differences (PCV + SB vs PCV  -SB), §: p < 0.05 for between-group differences (HCl vs OA-ALI) (*post hoc* Tukey multiple comparison test).

Table S5: Ventilation-Perfusion distributions

|  |  |  | BL ALI | Treatment | Time | Injury type | Mode | Inter-action |
| --- | --- | --- | --- | --- | --- | --- | --- | --- |
| RSS | HCl | + | 0.6 ± 0.6 | 0.3 ± 0.2 |  |  |  |  |
|  |  | - | 0.6 ± 0.4 | 0.6 ± 0.4 |  |  |  |  |
|  | OA | + | 1.1 ± 0.5 | 0.6 ± 0.4 |  |  |  |  |
|  |  | - | 1.1 ± 0.8 | 06.6 ± 0.4 |  |  |  |  |
| 0.005<$\dot{V}_{A}/\dot{Q}$<0.1  [%Q_T_] | HCl | + | 0.3 ± 0.9 | 11.0 ± 11.0 | * | * |  | * TI, TM, TMI |
|  |  | - | 3.5 ± 6.0 | 4.3 ± 5.3 |  |  |  |  |
|  | OA | + | 0.4 ± 1.2 | 1.8 ± 4.0 |  |  |  |  |
|  |  | - | 0.7 ± 1.7 | 1.2 ± 2.8 |  |  |  |  |
| 0.1<$\dot{V}_{A}/\dot{Q}$<10  [%Q_T_] | HCl | + | 72.0 ± 11.6 | 65.1 ± 19.4 | * |  |  | ** TM |
|  |  | - | 68.3 ± 13.3 | 57.7 ± 24.4 # |  |  |  |  |
|  | OA | + | 66.7 ± 23.9 | 71.9 ± 31.6 |  |  |  |  |
|  |  | - | 66.7 ± 24.9 | 51.4 ± 20.5 # |  |  |  |  |
| 10<$\dot{V}_{A}/\dot{Q}$<100  [%Ve] | HCl | + | 4.0 ± 4.9 | 7.1 ± 7.1 |  | * |  |  |
|  |  | - | 4.0 ± 3.5 | 4.9 ± 4.4 |  |  |  |  |
|  | OA | + | 2.7 ± 4.2 # | 2.2 ± 0.4 # |  |  |  |  |
|  |  | - | 1.6 ± 2.0 # | 1.0 ± 1.3 # |  |  |  |  |
| Mean(Q) | HCl | + | 1.42 ± 0.15 | 0.67 ± 0.28 + | * |  |  | * TI, TM, TMI |
|  |  | - | 1.1 ± 0.4 | 0.95 ± 0.34 |  |  |  |  |
|  | OA | + | 1.20 ± 0.26 | 1.04 ± 0.36 |  |  |  |  |
|  |  | - | 1.12 ± 0.29 | 0.89 ± 0.11 |  |  |  |  |
| Log SD_Q_ | HCl | + | 0.85 ± 0.20 | 1.55 ± 0.44 | * |  |  | * TM |
|  |  | - | 1.08 ± 0.47 | 1.35 ± 0.61 |  |  |  |  |
|  | OA | + | 0.90 ± 0.27 | 0.9 ± 0.52 |  |  |  |  |
|  |  | - | 0.83 ± 0.29 | 1.02 ± 0.42 |  |  |  |  |
| Mean(V_A_) | HCl | + | 2.88 ± 0.65 | 3.42 ± 1.25 |  |  |  |  |
|  |  | - | 2.69 ± 0.51 | 3.03 ± 1.01 |  |  |  |  |
|  | OA | + | 2.57 ± 0.50 | 2.45 ± 1.30 |  |  |  |  |
|  |  | - | 2.16 ± 0.52 | 1.87 ± 0.89 |  |  |  |  |
| Log SD_V_ | HCl | + | 0.78 ± 0.14 | 0.99 ± 0.25 |  |  |  | * TMI |
|  |  | - | 0.84 ± 0.14 | 0.85 ± 0.12 + |  |  |  |  |
|  | OA | + | 0.84 ± 0.19 | 0.78 ± 0.14 |  |  |  |  |
|  |  | - | 0.78 ± 0.15 | 0.81 ± 0.13 |  |  |  |  |

HCl: hydrochloric acid-induced acute lung injury (ALI), OA: oleic acid-induced ALI. SB: spontaneous breathing (+: pressure controlled ventilation with maintained spontaneous breathing, -: pressure controlled ventilation without SB). Interaction factors: T: time, M: mode, I: injury type.

RSS: residual sum of squares, Mean(Q): normal mean of perfusion, Mean(V_A_): normal mean of ventilation, Log SD_Q_: logarithmic standard deviation of perfusion, Log SD_V_: logarithmic standard deviation of ventilation.

*Post hoc* testing was always performed if a significant F ratio for a factor or the interaction of factors was obtained by repeated-measures analysis of variance (*: p < 0.05), but only significant differences are marked: +: p < 0.05 for within-group differences (ALI vs. Tr) and #: p < 0.05 for between-group differences (PCV + SB vs. PCV - SB), §: p < 0.05 for between-group differences (HCl vs. OA-ALI) (*post hoc* Tukey multiple comparison test).


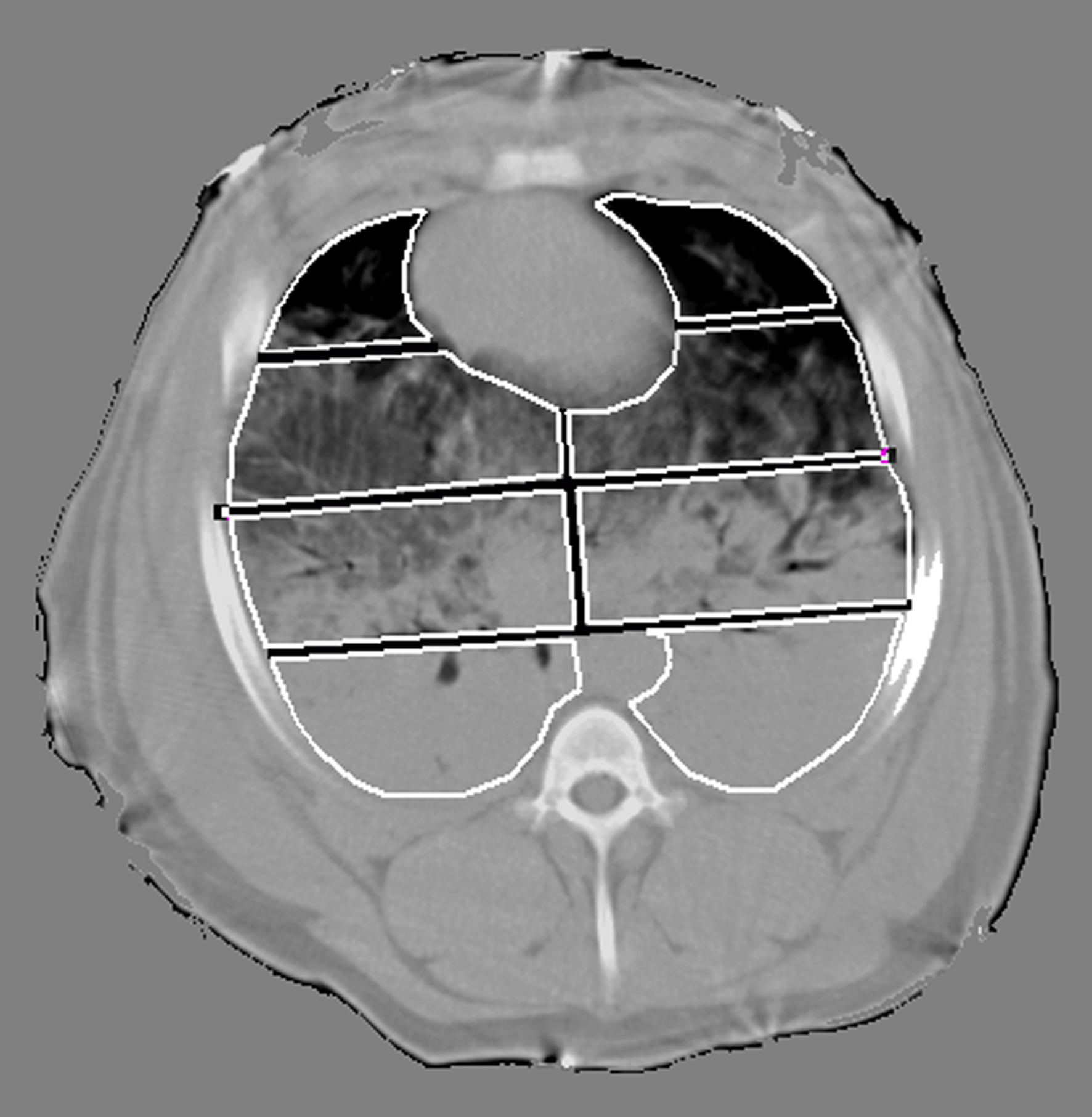


Figure S1: Regions of Interest (ROI) for evaluation of density distribution heterogeneities


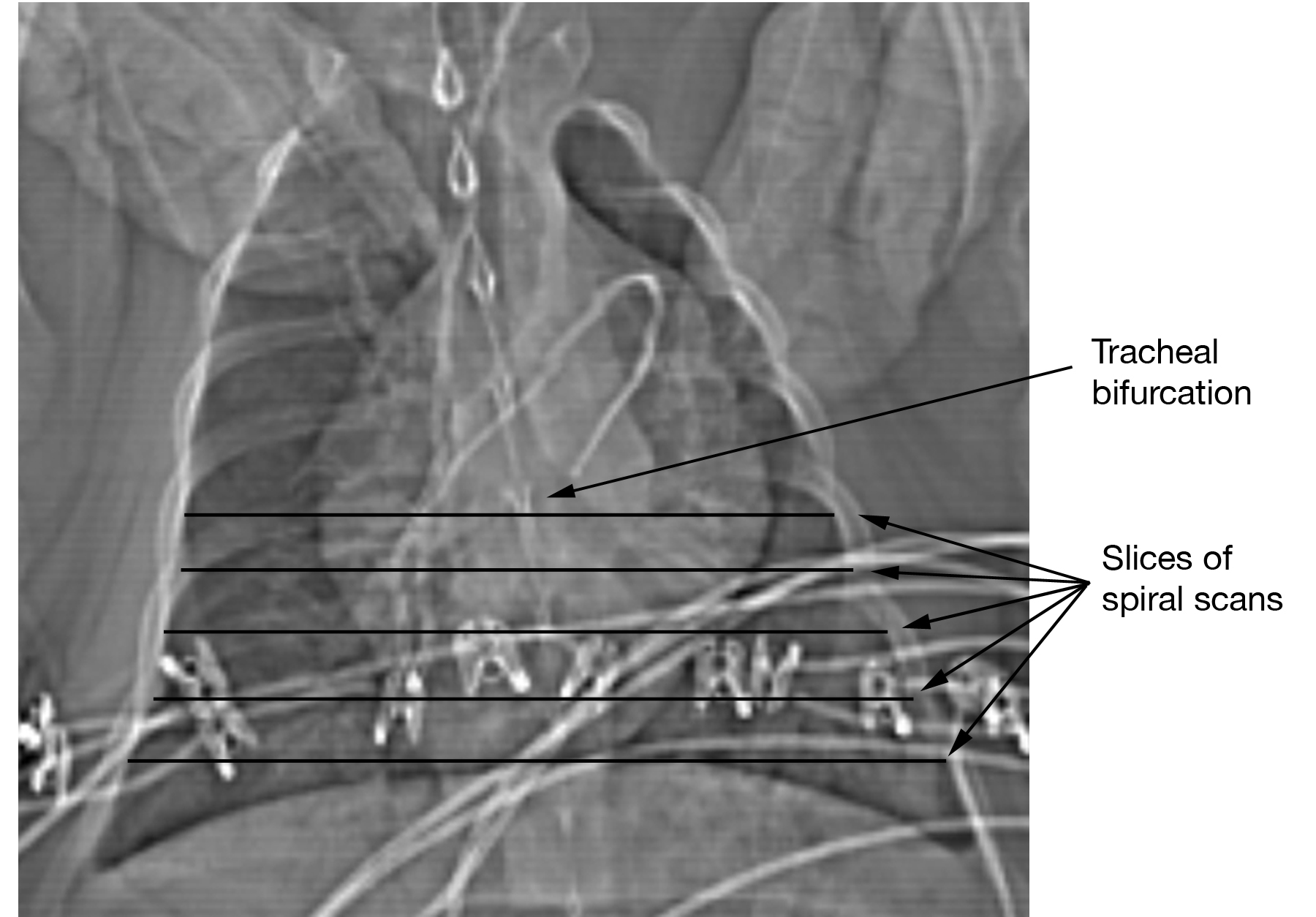


Figure S2: Slices from spiral scans for analysis of density distribution heterogeneities.
